# Predicting unplanned admissions to hospital in older adults using routinely recorded general practice data: development and validation of a prediction model

**DOI:** 10.3399/BJGP.2023.0350

**Published:** 2024-08-20

**Authors:** Jet H Klunder, Martijn W Heymans, Iris van der Heide, Robert A Verheij, Otto R Maarsingh, Hein PJ van Hout, Karlijn J Joling

**Affiliations:** Department of General Practice, Amsterdam UMC, Vrije Universiteit Amsterdam; Aging and Later Life, Amsterdam Public Health Research Institute, Amsterdam.; Department of Epidemiology and Data Science, Amsterdam UMC, Vrije Universiteit; Methodology, Amsterdam Public Health, Public Health Research Institute, Amsterdam.; Netherlands Institute for Health Services Research (NIVEL); Department of Languages, Literature and Communication, Faculty of Humanities, Utrecht University, Utrecht.; NIVEL, Utrecht; Tranzo, Tilburg School of Social and Behavioral Sciences, Tilburg University, Tilburg.; Department of General Practice, Amsterdam UMC, Vrije Universiteit Amsterdam; Aging and Later Life, Amsterdam Public Health Research Institute, Amsterdam.; Department of General Practice, Amsterdam UMC, Vrije Universiteit Amsterdam; Aging and Later Life, Amsterdam Public Health Research Institute, Amsterdam.; Department of Medicine for Older People, Amsterdam UMC, Vrije Universiteit Amsterdam; Aging and Later Life, Amsterdam Public Health Research Institute, Amsterdam.

**Keywords:** dementia, general practice, older adults, prediction model, primary care, unplanned admissions to hospital

## Abstract

**Background:**

Unplanned admissions to hospital represent a hazardous event for older people. Timely identification of high-risk individuals using a prediction tool may facilitate preventive interventions.

**Aim:**

To develop and validate an easy-to-use prediction model for unplanned admissions to hospital in community-dwelling older adults using readily available data to allow rapid bedside assessment by GPs.

**Design and setting:**

This was a retrospective study using the general practice electronic health records of 243 324 community-dwelling adults aged ≥65 years linked with national administrative data to predict unplanned admissions to hospital within 6 months.

**Method:**

The dataset was geographically split into a development (*n* = 142 791/243 324, 58.7%) and validation (*n* = 100 533/243 324, 41.3%) sample to predict unplanned admissions to hospital within 6 months. The performance of three different models was evaluated with increasingly smaller selections of candidate predictors (optimal, readily available, and easy-to-use models). Logistic regression was used with backward selection for model development. The models were validated internally and externally. Predictive performance was assessed by area under the curve (AUC) and calibration plots.

**Results:**

In both samples, 7.6% (development cohort: *n* = 10 839/142 791, validation cohort: *n* = 7675/100 533) had ≥1 unplanned hospital admission within 6 months. The discriminative ability of the three models was comparable and remained stable after geographic validation. The easy-to-use model included age, sex, prior admissions to hospital, pulmonary emphysema, heart failure, and polypharmacy. Its discriminative ability after validation was AUC 0.72 (95% confidence interval = 0.71 to 0.72). Calibration plots showed good calibration.

**Conclusion:**

The models showed satisfactory predictive ability. Reducing the number of predictors and geographic validation did not have an impact on predictive performance, demonstrating the robustness of the model. An easy-to-use tool has been developed in this study that may assist GPs in decision making and with targeted preventive interventions.

## Introduction

Increasing rates of unplanned admissions to hospital in older adults are a major burden on healthcare systems worldwide. For patients, unplanned hospital admissions are associated with functional decline and reduced quality of life.^1^ People with dementia are at particularly high risk of unplanned admissions, associated with worsening of pre-existing cognitive problems and an increased risk of readmission and death.^2^^–^^4^

Preventing unplanned admissions is critical to ensure patient safety and wellbeing, and aligns with the World Health Organization’s philosophy of providing tailored care in appropriate settings for older adults.^5^ A proactive approach optimises the allocation of scarce healthcare resources and addresses a pervasive concern in healthcare systems worldwide, where increasing demand outpaces the capacity of healthcare professionals. In the Netherlands, the integral care agreement (ICA) of 2022 prioritises preventive measures for acute care, particularly for older adults. Through education, prevention, and early signalling initiatives, the ICA aims to reduce unplanned admissions to hospital.^6^

Interventions such as providing an anticipatory care plan, telemedicine, and integrating a multidisciplinary geriatric team have been shown to reduce the number of unplanned admissions to hospital.^7^^–^^10^ However, timely identification of high-risk groups is essential for implementing proactive and targeted interventions.

**Table:** How this fits in

Unplanned hospital admissions in older adults are a critical concern for patients, family caregivers, healthcare professionals, and service planners. In this study a robust and easy-to-use prediction model has been developed and validated using routinely recorded data from general practices to predict the risk of unplanned hospital admissions in community-dwelling older adults. Identifying older adults at high risk can facilitate targeted preventive interventions, such as case management, telemedicine, or anticipatory care planning. Moreover, the model could also be utilised by policymakers for capacity planning of hospital beds.

GPs are patients’ primary point of contact and act as gatekeepers in many healthcare systems, such as the Netherlands.^11^ Therefore, they play a pivotal role in identifying those at risk for unplanned admission to hospital and the targeting of preventive interventions. A prediction model that can accurately predict high-risk individuals by reusing patient registration data could help GPs identify these individuals. The use of electronic health record (EHR) data offers opportunities for the development, integration, and automated calculation of an individual’s risk, because it contains comprehensive patient information and is derived from routine health care. The utilisation of these readily available data for the development of a prediction model facilitates ease of use and reduces time burden on GPs. Previous research has shown that administrative data can be useful in accurately predicting unplanned admissions to hospital.^12^^,^^13^ However, the methodological quality of these studies was limited and many models required additional data collection, making clinical use difficult. Models based on routine care data have a lower threshold and might therefore be used more frequently. As a result, their potential impact would be greater, even if the predictive power is similar.

The aim of this study was to develop and validate a practical and easy-to-use prediction model for unplanned admissions to hospital using a Dutch representative sample of older people in general practice. The model was developed using current state-of-the-art methods and incorporating readily available EHR data complemented with national administrative data. Also, the study specifically assessed the predictive performance of the model in a subsample of individuals with cognitive decline or dementia.

## Method

This study is reported according to the Transparent Reporting of a multivariable prediction model for Individual Prognosis or Diagnosis (TRIPOD) guidelines.^14^

### Sources of data

Pseudonymised EHR data from GPs linked with data from national administrative databases were used in this study. The baseline data covered the year 2013; outcomes were assessed in 2014. The routine EHRs used were from a nationally representative sample of 417 Dutch general practices participating in NIVEL-Primary Care Database (NIVEL-PCD).^15^ This database covers about 10% of the Dutch population and is representative in terms of practice type, urbanisation level, and age and gender distribution.^15^^,^^16^ Data include information on chronic conditions, medication, and GP consultations. GPs receive support to assist them with coding and also feedback about the quality of their recording.^16^ In the Netherlands, all Dutch inhabitants are registered with a GP and have mandatory health insurance. GP care is fully insured, therefore the threshold for consultation is low. Nine out of ten people aged ≥65 years visit their GP at least once a year, with an average of eight consultations per year.^17^

Administrative data were provided by Statistics Netherlands, the governmental institution responsible for processing statistical data in the Netherlands. These included demographic information and data on admissions to long-term care facilities and death. Data on admissions to hospital were derived from the Dutch Hospital Data (DHD) database, made available by Statistics Netherlands. In 2013 and 2014, DHD contained data from 87 out of 88 general and academic hospitals in the Netherlands.

### Study population

**Figure 1.**
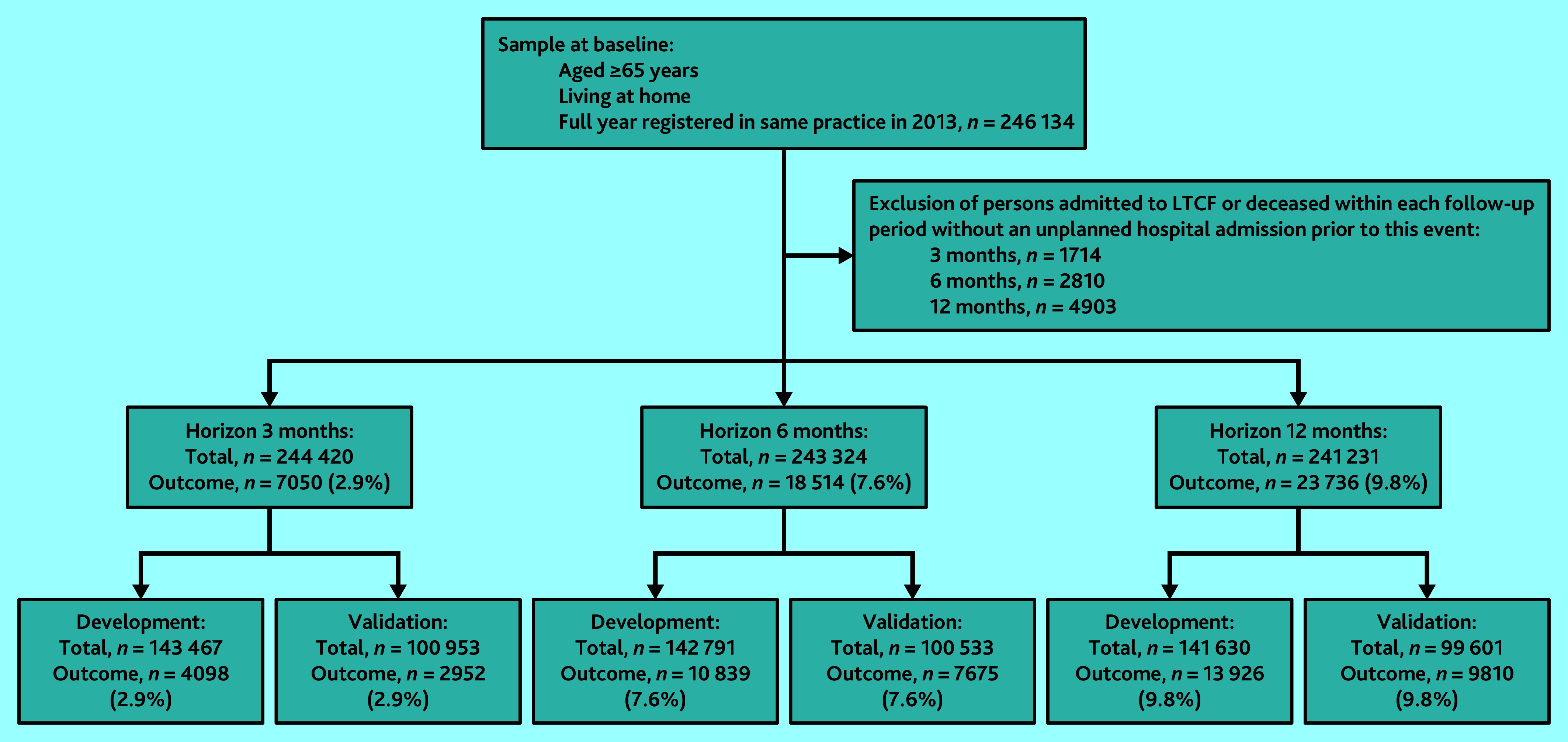
Flow of participants through study. LTCF = long-term care facility.

The study population consisted of individuals aged ≥65 years, living at home, and registered uninterrupted in one practice between 1 January 2013 and 31 December 2013 (baseline period). To avoid potential noise from admissions to a long-term care facility and from deaths in predicting the outcome, individuals who did not experience an unplanned admission among those who died or were admitted to a long-term care facility within the prediction period were excluded from the analysis (see Supplementary Figure S1). The number of excluded individuals varied depending on the follow-up period (3, 6, and 12 months) (Figure 1).

### Outcome

The primary outcome was unplanned hospital admissions with ≥1 overnight stay within 6 months and derived from national administrative data. Admissions were defined as unplanned when immediate treatment or assistance within 24 h was necessary according to the medical specialist.^18^ Admissions without an overnight stay and admissions for psychiatric conditions were excluded as these often require different care trajectories. Secondary outcomes were unplanned admissions within 3 and 12 months.

### Predictors

Updating existing prediction models was not feasible because of the incomparability of the predictors in this study’s dataset with the predictors in existing models, as well as the low methodological quality of these studies.^12^ To (partially) incorporate information from existing models, in the current study variables commonly included in existing models were selected as candidate predictors for the model, for example, prior admissions to hospital and several chronic conditions.^12^ In addition, variables were selected based on the insights from a focus group study that was organised among primary healthcare professionals (to be published) and based on the clinical expertise of the authors.

Ultimately, 29 candidate predictors were selected including age, sex, migration background, income, living situation, chronic conditions, prescription medications, and healthcare utilisation (see Supplementary Table S1 for a detailed description). Chronic conditions were derived from World Health Organization International Classification of Primary Care (ICPC-1)^19^ coded EHR data recorded up to the end of the baseline period. In NIVEL-PCD, GPs received feedback on the quality of recording and support to assist them with coding.^16^ Chronic conditions were selected because of their high prevalence in older adults.^20^ Dementia was added because of its strong association with admissions to hospital.^2^ Medication variables were derived from prescription data coded with the Anatomical Therapeutic Chemical classification system and included when a medication was prescribed in a chronic fashion (that is, >2 prescriptions^21^) in the year before baseline. Consult declarations (CTG-codes in Dutch) were derived from coded claims data recorded in general practices in the year before baseline.

### Missing data

As the data were derived from routine care processes, any undocumented information in the EHR was not indicated. For the data provided by Statistics Netherlands, income data had missing values for 116 individuals (<0.01%). This justified conducting a complete case analysis considering the negligible proportion of missing data and the minimal potential impact on the results.^12^^,^^13^^,^^22^^,^^23^

### Statistical analysis

Linearity was assessed for continuous variables using restricted cubic splines.^24^ Non-linear variables were tested as splines and as categorical variables in the logistic model. If the spline did not improve performance, the categorical variant was chosen because the authors wanted this to be a practical model. Collinearity was evaluated using variance inflation factors (VIFs). VIFs ranged between 1.01 and 2.43, therefore problematic collinearity was absent.^25^

#### Model development

The large sample provided sufficient statistical power to split the sample into a development and validation sample based on geographic region. The larger sample, that is, the six southernmost provinces (*n* = 142 791/243 324, 58.7%), was used for development and the smaller sample for validation (see Supplementary Figure S2). Geographic validation is considered a stronger approach compared with a random split sample procedure.^14^^,^^26^

For model building, this study followed the recommended steps outlined in the TRIPOD guidelines^14^ and by Steyerberg.^27^ Multivariable logistic regression with backward stepwise selection (*P*<0.01) was performed using all 29 candidate predictors to design an optimal model. Given the sample size, there was sufficient power to fit a more parsimonious model by incrementally removing the variables with weakest association, until the area under the curve (AUC) deteriorated by ≥0.01. Internal validation was performed through bootstrapping (*n* = 250).

This procedure was repeated twice with smaller subsets of candidate predictors to develop a model with only variables readily available from the EHR (readily available model) and with only easy-to-use variables (easy-to-use model), using 24 and 22 candidate predictors, respectively (see Supplementary Table S1). The easy-to-use model was designed to allow rapid completion by a GP; variables were therefore selected that are quick and easy to fill. All three models were validated in the northern sample.

#### Model performance

Discrimination was evaluated through AUC and calibration through calibration plots, intercept, and slope. The shrinkage factor was determined to quantify overfitting. Classification measures were also assessed, including sensitivity, specificity, and positive and negative predictive values, for multiple probability thresholds. The optimal probability threshold was determined using the Youden index.^28^

#### Sensitivity analysis

Sensitivity analyses were undertaken in the optimal model to assess performance for different follow-up periods (3 and 12 months). Furthermore, performance was assessed in subsamples of people with cognitive decline or dementia (ICPC-1 P20 or P70). Additionally, model performance was evaluated in a sample including individuals who had died or been admitted to a long-term care facility within 6 months. Statistical analyses were performed using IBM SPSS Statistics (version 26) and R Studio (version 4.1.2) using packages *rms*, *pROC*, and *psfmi.*

## Results

### Participants

Overall, 243 324 individuals were included in the 6-month sample (development *n* = 142 791/243 324, 58.7% and *n* = 100 533/243 324, 41.3% validation sample) (Figure 1). Prevalence of candidate predictors and incidence of outcome are shown in Table 1. In both samples, median age was 72 years (interquartile range [IQR] 68–78); in the development and validation samples, 53.8% (*n* = 76 892/142 791) and 53.9% (*n* = 54 141/100 533) were female, and approximately 41.9% (*n* = 59 867/142 791) and 43.3% (*n* = 43 534/100 533) had ≥2 comorbidities, respectively, of which osteoarthritis was most prevalent. In total, 12.8% (*n* = 18 292/142 791) experienced ≥1 admission to hospital in the year before baseline in the development sample. In both samples, 7.6% (development cohort: *n* = 10 839/142 791, validation cohort: *n* = 7675/100 533) experienced ≥1 unplanned admissions to hospital within 6 months.

**Table 1.** Characteristics of candidate predictors in 6-month development and validation samples

**Variable**	**Development (South) (*n* = 142 791)**	**Validation (North) (*n* = 100 533)**
**Age, years, median (IQR)**	72 (68–78)	72 (68–78)

**Sex, female**	76 892 (53.8)	54 141 (53.9)

**Living alone**	44 104 (30.9)^a^	32 720 (32.5)^a^

**Recent widowhood**	11 073 (7.8)	7885 (7.8)

**Non-Western migration background**	4065 (2.8)^a^	3587 (3.6)^a^

**Annual household income above €25 500^b^**	51 913 (36.4)^a^	34 922 (34.7)^a^
**Admissions in past year**		
1	13 034 (9.1)^a^	9548 (9.5)^a^
≥2	5258 (3.7)^a^	3926 (3.9)^a^

**Cancer**	24 637 (17.3)^a^	18 341 (18.2)^a^

**Coronary artery disease**	19 231 (13.5)	13 592 (13.5)

**Heart failure**	5796 (4.1)^a^	4617 (4.6)^a^

**Stroke (ischaemic or haemorrhage) or TIA**	10 604 (7.4)	7393 (7.4)

**Chronic neck or back disorder**	16 459 (11.5)	11 777 (11.7)

**Osteoarthritis**	27 661 (19.4)^a^	20 124 (20.0)^a^

**Anxiety disorder**	2805 (2.0)	2081 (2.1)

**Depression**	4898 (3.4)	3524 (3.5)

**COPD**	12 908 (9.0)	9286 (9.2)

**Diabetes mellitus**	23 521 (16.5)^a^	17 423 (17.3)^a^

**Dementia**	1910 (1.3)	1365 (1.4)

**Multimorbidity**	59 867 (41.9)^a^	43 534 (43.3)^a^

**Polypharmacy**		
5–9 prescription medication	29 951 (21.0)^a^	22 058 (21.9)^a^
≥10 prescription medication	4898 (3.4)^a^	3471 (3.5)^a^

**Blood thinners (VKA, DOAC, or antiplatelet)**	41 620 (29.1)^a^	28 643 (28.5)^a^

**FRIDs, median (IQR)**	0 (0–1)	0 (0–1)

**Taking ≥2 FRIDs and ≥1 blood thinner**	8733 (6.1)	6191 (6.2)

**NSAIDs**	7666 (5.4)	5266 (5.2)

**‘Triple whammy’^c^**	2473 (1.7)	1835 (1.8)

**CTV contacts with GP or practice nurse past year, median (IQR)**	5 (2–9)	5 (2–9)

**Percentage home visits past year**		
None (reference)	116 664 (81.7)^a^	81 542 (81.1)^a^
Up to 25%	13 695 (9.6)^a^	9826 (9.8)^a^
>25%	12 432 (8.7)^a^	9165 (9.1)^a^

**Change in contact rate in past 3 months**		
Fewer than previous 3 months (reference)	66 683 (46.7)^a^	46 011 (45.8)^a^
The same as previous 3 months	41 292 (28.9)^a^	29 869 (29.7)^a^
More than previous 3 months	34 816 (24.4)^a^	24 653 (24.5)^a^

**Possible care avoider^d^**	6427 (4.5)	4529 (4.5)

**Unplanned hospital admission within 6 months**	10 839 (7.6)	7675 (7.6)

*Data are* n *(%) unless otherwise specified.*

a P*<0.05 between groups.*

b *Annual household income had 83 missing in the southern sample and 33 missing in the northern sample.*

c *Concurrent use of an antidiuretic, ACE inhibitor, and NSAID (see Supplementary Table S1).*

d *Defined as ≥1 chronic condition and no registered contact with GP or practice nurse in the past year (see Supplementary Table S1). ACE = angiotensin-converting enzyme. COPD = chronic obstructive pulmonary disease. CTV = consultations, telephone consultations, and home visits. DOAC = direct oral anticoagulants. FRIDs = fall risk increasing drugs. IQR = interquartile range. NSAID = non-steroidal anti-inflammatory drug. TIA = transient ischaemic attack. VKA = vitamin K antagonists.*

### Model development and validation

**Figure 2.**
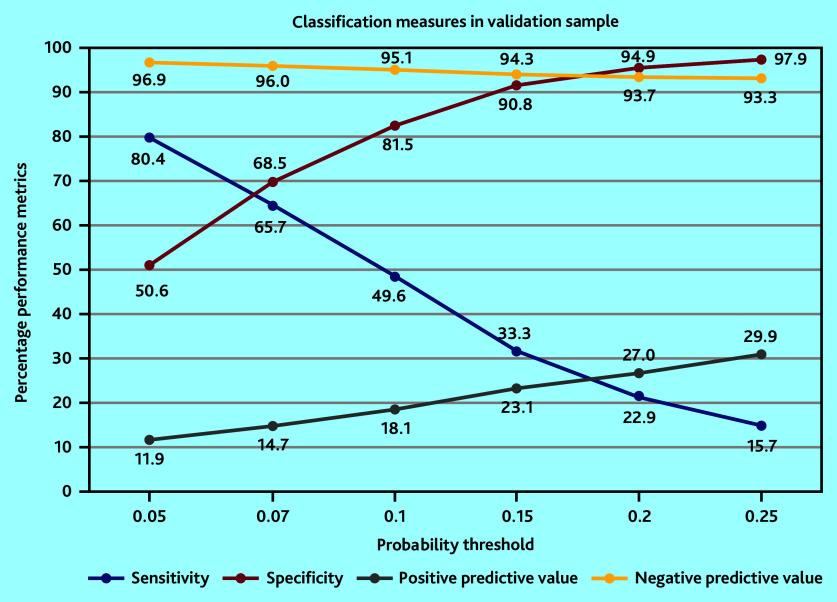
Graphical presentation of performance measures of the optimal model in the validation sample.

The optimal model included eight predictors: sex, age, prior admissions to hospital, chronic obstructive pulmonary disease (COPD), polypharmacy, use of blood thinners, number of GP or practice nurse consultations, and percentage of home visits compared with all contacts with a GP (Table 2). When applied to the validation sample, the AUC was 0.73 (95% confidence interval [CI] = 0.72 to 0.73). Youden’s optimal probability threshold was 0.07, reflecting a sensitivity of 65.7% and a specificity of 68.5% in the validation sample (Table 3 and Figure 2). Performance measures are reported for multiple probability thresholds to accommodate varying clinician preferences for risk estimation.

**Table 2.** The final prediction models from the multivariable logistic regression based on the development sample together with OR (95% CI) and the AUC in the development and validation samples

**Intercept, predictors, AUC**	**Optimal model**				**Readily available model**				**Easy-to-use model**			

	**β coefficient (95% CI)**		**OR**	***P*-value**	**β coefficient (95% CI)**		**OR**	***P*-value**	**β coefficient (95% CI)**		**OR**	***P*-value**
**Intercept**	−6.036	(−6.263 to −5.801)			−5.627	(−5.851 to −5.404)			−6.784	(−6.996 to −6.571)		

**Age, years**	0.040	(0.037 to 0.043)	1.04	<0.001	0.035	(0.032 to 0.038)	1.04	<0.001	0.053	(0.050 to 0.055)	1.05	<0.001

**Sex, female**	−0.279	(−0.321 to −0.237)	0.76	<0.001	−0.330	(−0.372 to −0.288)	0.72	<0.001	−0.233	(−0.274 to −0.192)	0.79	<0.001

**Admissions in past year**				<0.001								<0.001
None (reference)	0		1						0		1	
1	0.499	(0.441 to 0.557)	1.65						0.660	(0.603 to 0.716)	1.93	
≥2	0.972	(0.897 to 1.046)	2.64						1.231	(1.160 to 1.302)	3.43	

**Heart failure**									0.378	(0.30 to 0.45)	1.46	<0.001

**COPD**	0.420	(0.363 to 0.478)	1.52	<0.001	0.430	(0.372 to 0.487)	1.54	<0.001	0.436	(0.378 to 0.493)	1.55	<0.001

**Polypharmacy**				<0.001				<0.001				<0.001
0–4 medications (reference)	0		1		0		1		0		1	
5–9 medications	0.380	(0.328 to 0.431)	1.46		0.424	(0.373 to 0.475)	1.53		0.574	(0.528 to 0.620)	1.78	
≥10 medications	0.613	(0.526 to 0.701)	1.85		0.715	(0.628 to 0.802)	2.0		1.012	(0.934 to 1.090)	2.75	

**Blood thinners**	0.214	(0.167 to 0.261)	1.24	<0.001	0.246	(0.199 to 0.293)	1.28	<0.001				

**Number of CTV contacts**	0.022	(0.019 to 0.025)	1.02	<0.001	0.028	(0.025 to 0.031)	1.03	<0.001				

**Percentage home visits**				<0.001				<0.001				<0.001
None (reference)	0		1		0		1					
≤25%	0.309	(0.246 to 0.372)	1.36		0.498	(0.437 to 0.558)	1.64					
>25%	0.470	(0.406 to 0.543)	1.60		0.641	(0.578 to 0.704)	1.90	<0.001				

**AUC (95% CI)**												
Development	0.73	(0.72 to 0.73)			0.72	(0.71 to 0.72)			0.72	(0.71 to 0.72)		
Validation	0.73	(0.72 to 0.73)			0.72	(0.71 to 0.72)			0.72	(0.71 to 0.72)		

*AUC = area under the curve. COPD = chronic obstructive pulmonary disease. CTV = consultations, telephone consultations, and home visits. OR = odds ratio.*

**Table 3.** Measures of predictive performance of the optimal model in the development and validation sample for multiple probability thresholds

**Probability threshold**	**Development**				**Validation**			
	**Sensitivity, %**	**Specificity, %**	**PPV, %**	**NPV, %**	**Sensitivity, %**	**Specificity, %**	**PPV, %**	**NPV, %**
**0.05**	79.7	51.2	11.8	96.8	80.4	50.6	11.9	96.9
**0.07**	64.5	69.8	14.9	96.0	65.7	68.5	14.7	96.0
**0.1**	48.3	82.6	18.6	95.1	49.6	81.5	18.1	95.1
**0.15**	31.1	91.5	23.2	94.2	33.3	90.8	23.1	94.3
**0.20**	20.6	95.4	26.9	93.6	22.9	94.9	27.0	93.7
**0.25**	14.2	97.4	31.0	93.3	15.7	97.9	29.9	93.3

*The 0.07 threshold is defined by Youden’s index as the optimal probability threshold. NPV = negative predictive value. PPV = positive predictive value.*

The readily available model contained all predictors of the optimal model except for prior admissions to hospital (Table 2). Compared with the optimal model, the AUC in the validation sample was marginally lower (AUC 0.72, 95% CI = 0.71 to 0.72).

The easy-to-use model included age, sex, admissions to hospital in the past year, heart failure, COPD, and polypharmacy (Table 2). When applied to the validation sample, this resulted in an AUC of 0.72 (95% CI = 0.71 to 0.72). To allow for individualised predictions of this model, a Microsoft Excel spreadsheet is provided as a supplement (see Supplementary Information S1).

For all three models, bootstrapping resulted in an optimism of the AUC, intercept, and slope <0.001, therefore no adjustments of the coefficients were required. Calibration of all models was good; the slope and intercept did not deviate to the extent that model updating was undertaken (see Supplementary Figure S3).

### Clinical implications of choice of cut-off value

Choosing a cut-off value provides the opportunity to stratify patients into low- and high-risk groups. This facilitates clinical decision making. To illustrate this, a practice consisting of 500 community-dwelling patients aged ≥65 years was considered. The consequences of two different cut-off values (or probability thresholds) were compared: 0.07 and 0.15. A prior probability of 7.6% for each patient (prevalence) was assumed. The 2 × 2 contingency tables for both cut-offs are shown (Tables 4 and 5).

**Table 4.** Threshold of 0.07: 2 × 2 contingency table

**Threshold = 0.07**	**Admission +**	**Admission −**	**Total**
**High risk**	25 (TP)	140 (FP)	165
**Low risk**	13 (FN)	322 (TN)	335
**Total**	38	462	500

*FN = false negative. FP = false positive. TN = true negative. TP = true positive.*

**Table 5.** Threshold of 0.15: 2 × 2 contingency table

**Threshold = 0.15**	**Admission +**	**Admission −**	**Total**
**High risk**	12 (TP)	39 (FP)	51
**Low risk**	26 (FN)	423 (TN)	449
**Total**	38	462	500

*FN = false negative. FP = false positive. TN = true negative. TP = true positive.*

Using a cut-off of 0.07 stratifies approximately one-third of the practice’s older population as high risk, requiring screening or intervention. However, this choice results in a high number of false positives, where individuals are identified as high risk but do not experience the predicted outcome. At the individual level, using a threshold of 0.07 increases the probability of a patient being classified as high risk for unplanned hospital admissions by a factor of 2, from 7.6% to 14.9%. This means that, out of 100 high-risk patients, 15 will have an unplanned hospital admission within 6 months.

Alternatively, using a cut-off value of 0.15, one in ten older patients will be classified as high risk, resulting in a substantially lower number of false positives. However, the number of false negatives doubles, indicating that some potential patients are missed. For a high-risk patient at the 0.15 threshold, the probability of unplanned hospital admissions increases by a factor of 3 to 23.2%. Consequently, out of 100 high-risk patients, 23 would experience an unplanned hospital admission within 6 months.

How much risk a clinician is willing to take to avoid missing an unplanned admission will depend on the clinician’s judgement. Opting for a lower threshold results in a low number of false negatives, but raises the probability of false positives, requiring a more extensive and labour-intensive screening process.

### Sensitivity analyses

Testing the optimal model in people with cognitive decline resulted in an AUC of 0.67 (95% CI = 0.65 to 0.69) in both samples. The optimal model showed good predictive ability when fitted in the 3- and 12-month follow-up samples. However, the calibration plots showed systematic over- and underestimation in the 3- and 12-month samples, respectively. Finally, evaluating the optimal model in a sample including those who died or were admitted to a long-term care facility within 6 months resulted in an AUC of 0.72 (95% CI = 0.72 to 0.73). See Supplementary Tables S2–S4 and Supplementary Figure S4 for details of these analyses.

## Discussion

### Summary

In this study, routinely recorded and linked health and census data were used to develop and validate an easy-to-use prediction model for unplanned admissions to hospital in community-dwelling older adults. Predictors associated with unplanned hospital admission included age, sex, admission to hospital in the past year, polypharmacy, the use of blood thinners, COPD, heart failure, number of consultations including telephone consultations and home visit contacts, and the percentage of home visits. The optimal model showed satisfactory discrimination and good calibration. Moreover, geographic validation, reducing the number of predictors, changing the prediction horizon, and including individuals who died or were admitted to long-term care facilities within the prediction period, all resulted in a negligible decrease in discriminative ability, demonstrating robustness of the model. This model lost discriminatory power in a subsample of individuals with dementia or cognitive decline.

These results should enable GPs to identify patients who may benefit from targeted admission prevention strategies. To improve predictions, the authors of the current study emphasise the importance of routine recording or incorporation of hospital admission data into the EHR.

### Strengths and limitations

A strength of this study is the use of multiple approaches for model development, providing valuable insights into relative effectiveness and practical utility. By considering the advantages and limitations of each approach, healthcare providers and policymakers can make informed decisions about which model is suitable for their specific needs and resources. The use of EHR data enriched with national administrative data resulted in the best predictive model, that is, the optimal model. Using structured EHR data allows the readily available model to be implemented nationwide. However, it includes more time-consuming variables compared with the easy-to-use model. By facilitating rapid bedside assessment, the easy-to-use model is more accessible to GPs, while incorporating the most predictive variable: prior admissions to hospital. Furthermore, the large longitudinal sample and its nationwide representativeness suggests these findings could be generalised across the Netherlands.

This study also has limitations. As advocated in the literature,^29^^,^^30^ updating an existing prediction model is preferred over simply developing a new model, so information from the previous models is not neglected. However, model updating is only valuable provided the original model’s development is appropriately performed, and variables and outcomes are determined in a similar way.^29^ For this study, however, the low quality of reporting in the previous studies,^12^ and the lack of several variables in the current dataset, made updating infeasible. Moreover, differences in care systems between countries complicate the transportability of existing models to other geographical populations,^31^ and no model had yet been developed in the Netherlands. Altogether, this large sample called for the development of a new model rather than updating an existing one. Nevertheless, to incorporate data from previous models as much as possible, the current study assessed the variables most frequently included in previous models as candidate predictors for inclusion in this model. Furthermore, although in this study the data are approximately 10 years old, the relevance remains. Reviews have shown the long-term trends and relative stability over time of included predictors of unplanned hospital admissions, such as prior healthcare use, chronic conditions, and polypharmacy.^12^^,^^13^^,^^32^

### Comparison with existing literature

Nineteen existing prediction models to predict admissions to hospital in older adults were identified.^12^ The current model showed similar performance and overlap in the most commonly included variables. However, whereas many existing models used a 12-month prediction horizon, in the current study a 6-month horizon was chosen from a clinical perspective because a high predicted probability of hospital admission within 6 months is more likely to trigger timely clinical action than the same probability of hospital admission within 12 months. However, model validation over 12 months demonstrated equivalent discriminatory ability, albeit with systematic underestimation, requiring adjustment of the intercept.

Two previous studies developed a model for people with dementia in primary care, and both demonstrated good predictive performance. These studies found (changes in) psychotropic medication, psychiatric diagnoses, and hypertension to be important predictors, along with previous admissions to hospital and polypharmacy.^33^^,^^34^ Other studies found duration and severity of dementia, caregiver burden, and continuity of care associated with hospital admissions.^35^^–^^37^ To improve predictive accuracy in individuals with cognitive decline, these predictors may need to be considered for inclusion in the model.

### Implications for research and practice

The implementation of ageing-in-place policies in the Netherlands in 2015, which included a reduction of the residential care capacity, has increased the number of older adults living in the community.^38^ As this study used data from before the implementation of this policy, a different case mix of community-dwelling older adults may be expected. Therefore, validation in more recent data is recommended. Additionally, the authors emphasise the importance of systematic recording of admissions to hospital in the EHR to enable practical implementation and to provide the most accurate risk estimates, as prior admissions to hospital is the strongest predictor of future admissions to hospital.

The models in the current study may support timely identification of and proactive interventions for older patients at risk of unplanned hospital admissions. When selecting the appropriate cut-off value for targeting interventions, clinicians should prioritise factors such as patient preference, intervention time burden, and the trade-off between intervention benefits and potential missed patients. Finally, the models could assist policymakers with estimating the required number of hospital beds in the region.

## Data

The data set used for this study is not publicly available, because it cannot leave the environment of Statistics Netherlands according to their privacy regulations. Requests to access the data set should be directed to the principal investigator of the BESIDE study (k.joling@amsterdamumc.nl).
